# Global preamplification simplifies targeted mRNA quantification

**DOI:** 10.1038/srep45219

**Published:** 2017-03-23

**Authors:** Thomas Kroneis, Emma Jonasson, Daniel Andersson, Soheila Dolatabadi, Anders Ståhlberg

**Affiliations:** 1 Sahlgrenska Cancer Center, Department of Pathology and Genetics, Institute of Biomedicine, Sahlgrenska Academy at University of Gothenburg, Medicinaregatan 1F, 413 90, Gothenburg, Sweden; 2 Institute of Cell Biology, Histology and Embryology, Medical University of Graz, Harrachgasse 21, 8010, Graz, Austria

## Abstract

The need to perform gene expression profiling using next generation sequencing and quantitative real-time PCR (qPCR) on small sample sizes and single cells is rapidly expanding. However, to analyse few molecules, preamplification is required. Here, we studied global and target-specific preamplification using 96 optimised qPCR assays. To evaluate the preamplification strategies, we monitored the reactions in real-time using SYBR Green I detection chemistry followed by melting curve analysis. Next, we compared yield and reproducibility of global preamplification to that of target-specific preamplification by qPCR using the same amount of total RNA. Global preamplification generated 9.3-fold lower yield and 1.6-fold lower reproducibility than target-specific preamplification. However, the performance of global preamplification is sufficient for most downstream applications and offers several advantages over target-specific preamplification. To demonstrate the potential of global preamplification we analysed the expression of 15 genes in 60 single cells. In conclusion, we show that global preamplification simplifies targeted gene expression profiling of small sample sizes by a flexible workflow. We outline the pros and cons for global preamplification compared to target-specific preamplification.

Technology improvements now allow for detection and quantification of small amounts of analytes, even individual molecules, in an accurate and quantitative manner. This enables clinical and scientific assessments of biomarkers in limiting sample types, including individual cells, liquid and tissue biopsies and cytological aspirates^1^,^2^,^3^,^4^,^5^. Today, gene expression profiling is typically performed using reverse transcription quantitative real-time PCR (RT-qPCR)^6^ or next generation sequencing (NGS)^7^. RT-qPCR is usually preferred if genes of interest are lowly expressed^8^, while NGS is favoured when a high number of genes or whole transcriptomes are to be assessed^9^.

To facilitate reliable quantification of multiple targets in small sample sizes, preamplification is a prerequisite. Several preamplification strategies exist, but most approaches can be defined to be either target-specific^10^,^11^,^12^ or global^13^,^14^. Target-specific preamplification is usually performed by multiplex PCR using predefined primer pools. Important parameters for successful target-specific preamplification include the use of low primer concentrations (10–20 times lower than for conventional PCR) in combination with extended annealing time (3 min or more) in a limited number of cycles (usually 20 cycles or less), allowing specific PCR products to be formed without introducing bias. In addition, high preamplification efficiencies are favoured, contra-intuitively, if the applied primer pool contain a high number of assays (≥96 assays) where well-optimised assays usually display efficiencies close to 100%^15^. However, some issues are related to target-specific preamplification: i) all individual assays need to be optimised for sensitivity and specificity in the multiplex PCR, ii) preparation of primer pools is time consuming, and iii) analysis of additional genes not part of the preamplification pool cannot be performed without additional preamplification of the initial sample, something which is usually not feasible due to low amount of sample. The use of a global preamplification approach can overcome these limitations, applying downstream targeted mRNA quantification. Global preamplification is target-independent and, thus, easy to standardise.

Here, we compared yield and reproducibility of global preamplification to that of target-specific preamplification for targeted mRNA quantification using downstream qPCR (Fig. 1a). To assess the overall performance of these preamplification strategies, we also monitored the reactions in real-time using SYBR Green I detection chemistry followed by melting curve analysis. Finally, to test the feasibility of applying global preamplification followed by targeted gene expression profiling, we analysed 60 single cells. Our data allow us to provide pros and cons for targeted mRNA expression profiling using global compared to target-specific preamplification approaches. Improved and simplified preamplification strategies will facilitate analysis of small sample sizes, including single cells.

## Results

**Figure 1:**
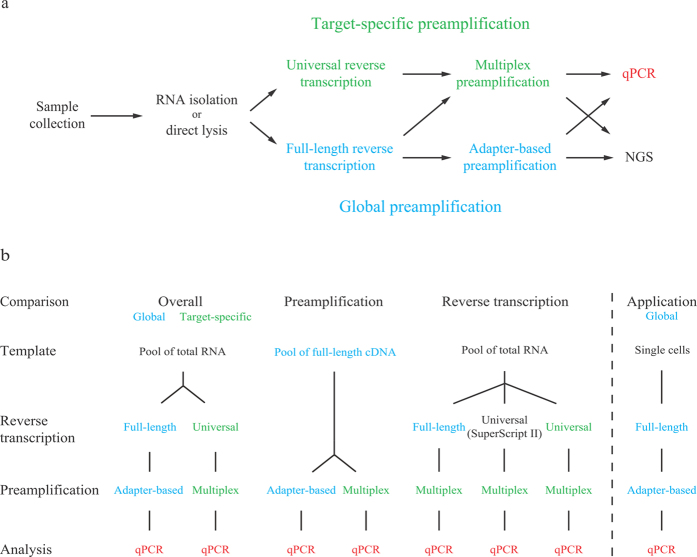
Preamplification strategies and experimental setup. (**a**) Isolated RNA or RNA of directly lysed cells can be preamplified by either global or target-specific preamplification and analysed with quantitative real-time PCR (qPCR) or next generation sequencing (NGS). In this study, we defined the properties of global preamplification in comparison to target-specific preamplification for targeted mRNA quantification using qPCR. To perform NGS analysis, the complete Smart-Seq2 protocol can be applied to globally preamplified cDNA^13^. (**b**) We assessed yield and reproducibility of the two preamplification strategies as outlined, including overall performance and that of the preamplification and reverse transcription (RT) steps separately. Total RNA isolated from MLS 2645–94 cells served as template for all yield and reproducibility tests. First, overall yield and reproducibility of global and target-specific preamplification were assessed performing full-length or universal RT using 100 pg total RNA per reaction (n = 5), respectively. cDNA corresponding to 30 pg total RNA of each reverse transcribed sample was preamplified (n = 1) and analysed by qPCR (n = 1, 96 assays). To evaluate preamplification, pooled cDNA obtained from full-length RT corresponding to 30 pg total RNA per reaction was used as template for adapter-based and multiplex preamplification (n = 4). Each preamplified sample was analysed by qPCR (n = 1, 96 assays). The effect of RT was assessed using 100 pg pooled total RNA per sample (n = 3), of which cDNA corresponding to 30 pg total RNA was multiplex preamplified (n = 1), and analysed by qPCR (n = 1, 96 assays). Finally, we demonstrated the feasibility of global preamplification by analysing single MLS 2645–94 cells (n = 60). Each cell was full-length reverse transcribed (n = 1), globally preamplified (n = 1) and analysed by qPCR (n = 1, 15 assays).

We applied the Smart-Seq2 protocol for global preamplification^13^. In the Smart-Seq2 approach each reverse transcribed RNA molecule containing a poly-A tail receives adapter sequences at its 5′ and 3′ ends (full-length RT). These two adapter sequences are designed to enable preamplification of all cDNA using a single primer (adapter-based preamplification). In comparison, target-specific preamplification is not dependent on the adapter primer. Here, an equimolar mixture of oligo-dT and random hexamers are preferably used to prime the reverse transcription (universal RT) to maximise the cDNA yield^16^. Resulting cDNA is then forwarded to multiplex preamplification using a pool of PCR primers, identical to those used in downstream qPCR. Details about the two preamplification strategies are shown in Supplementary Fig. S1. Here, we applied a defined set of 96 optimised assays (see Supplementary Table S1)^15^, comparing yield and reproducibility of global preamplification to that of target-specific preamplification using qPCR (Fig. 1a). In addition, we used pooled RNA and cDNA to compare yield and reproducibility of the different enzymatic steps in global and target-specific preamplification as outlined in Fig. 1b.

### Real-time monitoring of preamplification reaction can be used to determine optimal number of amplification cycles

**Figure 2:**
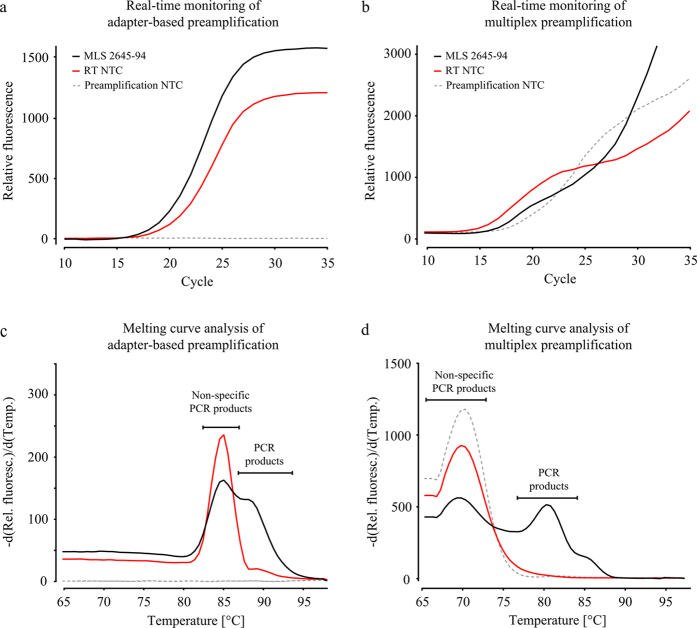
Real-time monitoring of preamplification. (**a**) Response curve for real-time monitoring of adapter-based preamplification using SYBR Green I detection chemistry following full-length reverse transcription (RT). (**b**) Response curve for real-time monitoring of multiplex preamplification using SYBR Green I detection chemistry following universal RT. cDNA corresponding to 30 pg total RNA isolated from MLS 2645–94 cells was used in each preamplification reaction. Melting curves for adaptor-based and multiplex preamplification are shown in (**c**) and (**d**), respectively. RT no-template controls (NTCs) and preamplification NTCs were included as references to distinguish specific from non-specific PCR products and to determine in what reactions these products were generated. –d(Rel. fluoresc.)/d(Temp.), –d(Relative fluorescence)/d(Temperature).

Preamplification should yield sufficient number of molecules for downstream analysis, while at the same time avoid introducing biases by cycling the reaction beyond its exponential phase. In several applications it is difficult to estimate the required number of preamplification cycles. To overcome this obstacle, we monitored the preamplification in real-time using SYBR Green I detection chemistry, which allowed us to determine the maximal number of preamplification cycles. We performed full-length and universal RT using 100 pg total RNA isolated from MLS 2645–94 cells. Figure 2a–d shows the amplification response curves and corresponding melting curves of adapter-based and multiplex preamplification using cDNA corresponding to 30 pg total RNA. The applied cDNA concentration is in the upper range of total RNA content reported for single mammalian cells^17^,^18^. RT no-template controls (NTCs) and preamplification NTCs were included as references to distinguish specific from non-specific PCR products and to determine in what reactions these products were generated. Compared to multiplex preamplification, the response curve for adapter-based preamplification showed up at higher Cq-values (Fig. 2a,b). Thus, to maximise the yield of adapter-based preamplification, we subsequently applied 24 cycles of preamplification. The corresponding number of cycles for multiplex preamplification was 20 (Fig. 2b), which is in agreement with other reports^15^,^19^. Both global and target-specific preamplification protocols generated non-specific PCR products in the RT NTCs, but the relative amount of non-specific PCR products was higher in target-specific preamplification (Fig. 2c,d). Furthermore, the NTC of adapter-based preamplification generated no amplification response curve, while the NTC of multiplex preamplification produced almost as much non-specific PCR products as in the RT NTC. Similar results were obtained with RNA from other cell lines (Supplementary Fig. S2).

### Global preamplification enables reproducible targeted mRNA quantification

**Figure 3:**
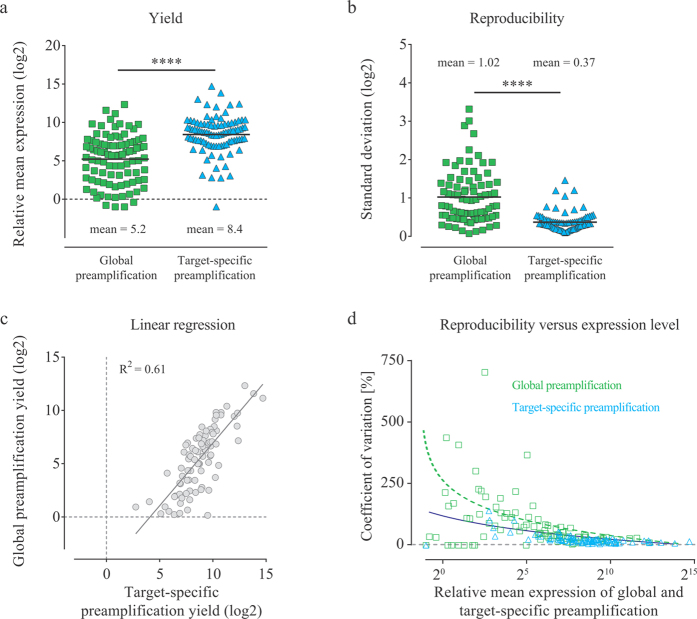
Comparison of global and target-specific preamplification. (**a**) Yield and (**b**) reproducibility of global compared to target-specific preamplification as outlined in Fig. 1b. Full-length (global) and universal (target-specific) reverse transcription were performed (n = 5) using 100 pg total RNA per reaction. cDNA corresponding to 30 pg total RNA per sample was used in the following preamplification step (n = 1) and 96 transcripts were subsequently analysed by targeted mRNA quantification using quantitative real-time PCR (n = 1). Each square (global) and triangle (target-specific) represents one assay. The horizontal solid bars indicate mean values and the horizontal dashed line indicates one molecule. ****Indicates p < 0.0001 using Wilcoxon matched-pairs signed rank test, n = 92. (**c**) Linear regression comparing the yields of global and target-specific preamplification. (**d**) Coefficient of variation versus mean expression. Each square (global) and triangle (target-specific) represents one assay. The curve fitting is to guide the eye only.

To test whether global preamplification generates adequate yield of molecules for downstream applications, we performed high-throughput qPCR on the platform that requires the highest template concentration, i.e., the 96.96 Dynamic Array on the BioMark system. We performed full-length and universal RT using 100 pg total RNA followed by adapter-based and multiplex preamplification with cDNA corresponding to 30 pg total RNA, respectively (Fig. 1b). Expression of 90 genes was detected applying global preamplification, while target-specific preamplification detected expression of 91 genes. Global preamplification generated 9.3-fold (p < 0.0001) lower yield and 1.6-fold (p < 0.0001) lower reproducibility than target-specific preamplification (Fig. 3a,b). The gene with largest expression difference between target-specific preamplification and global preamplification was *TGFB1*, where target-specific preamplification generated 660 times more molecules. *CDC25B* was the only gene which showed higher yield using global preamplification than with target-specific preamplification (Supplementary Fig. S3). Figure 3c shows how the yield of all genes correlates to each other applying global and target-specific preamplification. Figure 3d shows how the preamplification reproducibility correlates with expression level.

### Full-length RT and adapter-based preamplification are less efficient than universal RT and multiplex preamplification

We assessed yield and reproducibility of the two preamplification protocols in detail by studying the RT and preamplification steps separately (Fig. 1b). First, we compared the yield and reproducibility of adapter-based to that of multiplex preamplification using a common pool of cDNA generated by full-length RT. Adapter-based preamplification showed 3.2-fold (p < 0.0001) lower yield and 1.7-fold (p < 0.0001) lower reproducibility than multiplex preamplification (see Supplementary Fig. S4a,b). Linear regression showed that the yield of adapter-based and multiplex preamplification correlated with each other (Supplementary Fig. S4c). The largest observed difference between adapter-based and multiplex preamplification was again observed for *TGFB1*, where multiplex preamplification generated 540 times more molecules. Four genes (*CDC25B, EWSR1, MDM4* and *PPARG*) generated >2 times more molecules using adapter-based preamplification, where the largest difference was observed for *CDC25B* with a 20-fold difference (Supplementary Fig. S3).

Next, we compared the yield and reproducibility of full-length RT to that of universal RT using a common pool of total RNA followed by multiplex preamplification (Fig. 1b). The major differences between full-length RT and universal RT are the choice of reverse transcriptase and applied RT primers. Full-length RT uses SuperScript II and an oligo-dT_30_VN primer with an adapter sequence, while universal RT uses SuperScript III and a mixture of an oligo-dT_15_ primer and random hexamers. To test the effect of the reverse transcriptase we also performed universal RT with SuperScript II. Full-length RT generated 2.5-fold lower yield (p < 0.0001), but similar reproducibility compared to universal RT (see Supplementary Fig. S4d,e). The use of SuperScript III instead of SuperScript II in universal RT generated 1.4-fold higher yield (p < 0.0001). Linear regression showed that the yield of full-length and universal RT correlated with each other (Supplementary Fig. S4f).

### Single-cell gene expression profiling is possible applying global preamplification and targeted mRNA quantification

**Figure 4:**
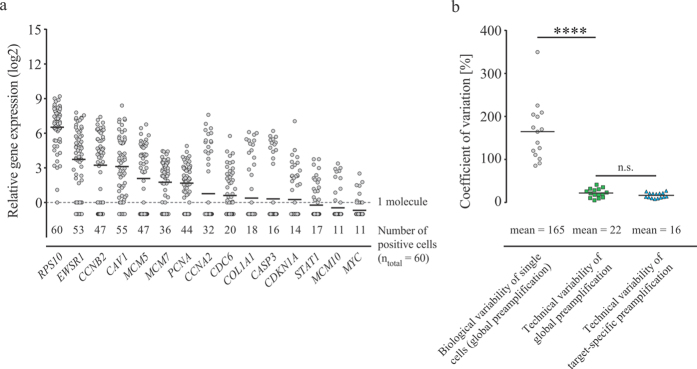
Single-cell gene expression profiling using global preamplification. (**a**) Sixty single cells were collected using fluorescence-activated cell sorting, globally preamplified (n = 1) and analysed by quantitative real-time PCR (n = 1). Relative expression is shown for each gene with every cell being represented by a circle. Number of cells expressing each gene is indicated. Horizontal bars indicate mean values and the dashed line indicates one molecule. (**b**) Biological versus technical variability. Biological coefficient of variation (CV) was calculated from single-cell data (n = 60), while technical CV was calculated on the same 15 assays using data in Fig. 3, applying global and target-specific preamplification on pooled total RNA (n = 5), respectively. The horizontal bars and values indicate mean CV values. ****Indicates p < 0.0001, n.s. not significant (p = 0.11), both using Wilcoxon matched-pairs signed rank test (n = 15).

To test the feasibility of global preamplification on limited biological samples, we performed full-length RT followed by adapter-based preamplification on 60 individual fluorescence-activated cell sorted cells and evaluated the preamplification products, analysing 15 genes by means of qPCR (Figs 1b and 4a). We included genes with low, intermediate and high expression levels (Supplementary Fig. S3). The number of cells expressing a particular gene correlated with their mean expression level. For example, the most abundantly expressed gene, *RPS10*, was expressed in all cells, while *MYC* and *MCM10* displayed the lowest mean expression levels and were only detected in 11 of 60 cells. Figure 4b shows that the biological variability among the individual cells is 7.5 times higher (p < 0.0001) than the technical variability of global preamplification. For these 15 assays, the technical variability of global preamplification was similar to that of the target-specific preamplification.

## Discussion

The need to use small sample sizes is rapidly increasing in many research fields, including clinical applications. Today, many analytes, such as DNA, RNA and protein, can accurately be detected and quantified, even in individual cells^13^,^20^,^21^,^22^,^23^,^24^. For mRNA analysis, NGS and qPCR are emerging as the two most commonly applied techniques. However, in order to analyse limited sample sizes, preamplification is generally required. The preamplification step can be bypassed when few genes, i.e. ≤10, intermediately or highly expressed, are to be quantified. When analysing only one gene, preamplification should be avoided^25^. One major limitation of most NGS and qPCR approaches is that they cannot easily be combined in order to benefit from each other. Here, we show that global preamplification used for library preparation in NGS can be applied to targeted mRNA quantification using qPCR, thereby simplifying the experimental workflow and enabling a variety of downstream applications. To determine the properties of global preamplification in targeted mRNA quantification, we compared it to target-specific preamplification in terms of yield and reproducibility using qPCR. For targeted mRNA quantification, we used an optimised 96 gene panel with preamplification and PCR efficiencies close to 100%^15^.

To enable reliable quantification, the preamplification yield should be maximised with maintained reproducibility. Hence, the number of preamplification cycles should be as high as possible, while the reaction is kept in its exponential phase to avoid introduction of bias. For small sample sizes it is usually not possible to determine RNA concentration and consequently it is difficult to estimate what number of preamplification cycles to apply. To overcome this bottleneck we monitored both global and target-specific preamplification in real-time using SYBR Green I detection chemistry. We used amplification response curves together with melting curve analyses to determine the maximum number of preamplification cycles that can be applied. We have previously shown that 20 cycles of target-specific preamplification is enough to provide sufficient molecules for reliable gene expression profiling of single cells performing high-throughput qPCR^15^. Here, we show that 24 amplification cycles are sufficient for processing cDNA corresponding to 30 pg total RNA using global preamplification. In both preamplification approaches the RT NTCs generated non-specific PCR products, while only multiplex preamplification NTCs displayed non-specific PCR products. This lack of non-specific PCR products in the adapter-based preamplification can be explained by the use of one single primer, while 96 primer pairs were used in multiplex preamplification.

Next, we assessed the overall yield and reproducibility of global preamplification (24 cycles) compared to target-specific preamplification (20 cycles). We detected expression by almost the same number of genes with both preamplification strategies, 90 and 91, respectively. However, the mean yield for the detected transcripts was 9.3-fold lower using global preamplification. By comparing the reproducibility versus expression level (Fig. 3d) we conclude that the superior reproducibility of target-specific preamplification was, at least in part, due to increased yield. Furthermore, the technical variability versus average expression level is relatively constant until lowly expressed genes are analysed.

The lower global preamplification yield was a result of decreased RT and adapter-based preamplification efficiencies. In universal RT we used a blend of oligo-dT and random hexamers to prime the reaction, while full-length RT was restricted to the use of oligo-dT. In addition, we used two different reverse transcriptases, SuperScript II and III. Our data show that both the priming strategy and the choice of reverse transcriptase affected the cDNA yield. We used SuperScript II in the full-length RT, since SuperScript III has been reported to generate lower yield of full-length cDNA with adapter sequences at both ends^13^,^26^. In target-specific preamplification, full-length cDNA is not required and our data indicate that universal RT with SuperScript III generated more cDNA molecules than SuperScript II, even if the two applied universal RT protocols are not identical. Furthermore, an optimal universal RT yield is generated when a blend of oligo-dT and random hexamers is used^16^,^27^. Our comparison of cDNA yields between full-length and universal RT is not taking into account how efficient the adapter sequence is introduced at the 3′ cDNA end by the template switching oligonucleotide, since our applied downstream multiplex preamplification is not cDNA adapter dependent. In addition, a decreased adapter-based preamplification efficiency is also expected, since long PCR products are generally amplified with lower efficiencies than shorter PCR products. Hence, the observed difference between adapter-based and multiplex preamplification can be explained by both incomplete template switching and low preamplification efficiency. Taking all this into account, our data suggest that global preamplification loses molecules at all steps compared to target-specific preamplification.

We demonstrated the feasibility of global preamplification by performing single-cell gene expression profiling, targeting 15 specific genes. By comparing the gene expression levels among individual cells we conclude that the biological variability was significantly higher than the technical variability of global preamplification. A consequence of applying global compared to target-specific preamplification in single-cell analysis is that, generally, more cells need to be analysed to compensate for the overall loss of yield and reproducibility.

The use of global over target-specific preamplification provides some significant advantages. Most importantly, samples processed with global preamplification can easily be forwarded to both targeted and global mRNA quantification. This allows samples to be initially screened by analysing the expression of a few genes (qPCR) and then further analysed in depth with additional target genes (qPCR), or at global level (NGS). Global preamplification is performed with the same protocol no matter what genes of interest will be selected for downstream analysis, while target-specific preamplification requires a new pool of primers for each application addressing new target genes. This, for example, is not compatible with commercial qPCR gene panels, since most manufacturers do not provide multiplex primer pools. Furthermore, multiplex PCR optimisation and primer pool preparations are time consuming. On the other hand, the global preamplification protocol is less sensitive and robust as shown by lower yield and reproducibility. Because of this, some lowly expressed genes may not be reliably quantified or even detected. To test the impact of reduced preamplification yield and its impact on defining subpopulations we made use of a publicly available data set generated with target-specific preamplification^28^. By dividing all molecule numbers with 9.3 and then eliminating all values below one, we mimicked the reduction in yield caused by global preamplification. Principal component analysis showed that we could separate subpopulations of single cells almost to the same extent after the molecule reduction (Supplementary Fig. S5). Thus, single-cell gene expression profiling using global preamplification is feasible as long as the gene panel is not restricted solely to lowly expressed genes. Another drawback of our global preamplification protocol is its restriction to full-length mRNA, which makes analyses of partially degraded samples, including formalin-fixed paraffin-embedded tissues, challenging. One possibility to handle samples with poor RNA integrity is to use a poly(A)-independent global preamplification strategy^29^.

**Figure 5:**
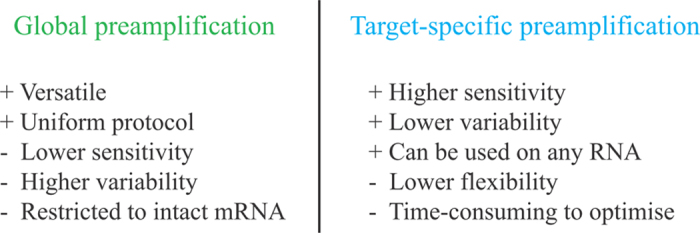
Pros and cons of global and target-specific preamplification.

In conclusion, we have shown that global preamplification simplifies gene expression profiling of small sample sizes, including single cells, by a flexible workflow. Global preamplification offers some advantages and disadvantages compared to target-specific preamplification (summarised in Fig. 5).

## Materials and Methods

### Cell cultures, extraction and single cell collection

Cell lines were cultured at 37 °C and 5% CO_2_. Myxoid liposarcoma (MLS) cell lines 402–91, 2645–94 and DL 221, as well as stable clones of fibrosarcoma cell line HT1080 expressing FUS-DDIT3-EGFP or EGFP^30^,^31^,^32^,^33^ were cultured in RPMI 1640 GlutaMax medium supplemented with 10% fetal bovine serum, 100 U/ml penicillin and 100 μg/ml streptomycin. All cell lines were passaged using 0.25% trypsin containing 0.5 mM EDTA. All cell culture reagents were obtained from Thermo Fisher Scientific.

Total RNA was extracted using the RNeasy Mini Kit including DNase treatment (Qiagen) according to the manufacturer’s recommendations and stored at −80 °C. High RNA integrity was confirmed using a 2100 BioAnalyzer Instrument (Agilent Technologies). For experiments, isolated total RNA was diluted in direct lysis buffer (1 μg/μl bovine serum albumin supplied in 2.5% glycerol, Thermo Fisher Scientific, and 0.2% Triton X-100, Sigma-Aldrich) to 5 μl containing 100 pg total RNA.

For single-cell sampling, MLS 2645–94 cells were trypsinised, washed once with 1x PBS (Thermo Fisher Scientific) and resuspended in 1x PBS supplemented with 2% bovine serum albumin (Sigma-Aldrich). Cell aggregates were removed using a 70 μm cell strainer (BD Biosciences). 7-aminoactinomycin D staining (BD Biosciences) was used to collect viable cells. Cell sorting was done as previously published^34^ with minor changes. In short, single cells were sorted into 96-well plates (Thermo Fisher Scientific) containing 5 μl direct lysis buffer using a BD FACSAria II instrument and the FACSDiva software (both BD Biosciences). Plates with cell lysates were placed on dry ice immediately after sorting and stored at −80 °C.

### Reverse transcription

The Smart-Seq2 protocol^13^ was used to generate full-length cDNA from total RNA and sorted single cells. Briefly, 1 μM adapter sequence-containing oligo-dT_30_VN (5′-AAGCAGTGGTATCAACGCAGAGTACT_30_VN-3′) and 1 mM dNTP (both Sigma-Aldrich) were incubated in 6.7 μl containing 100 pg total RNA, single cells or no cells (RT no-template control) at 72 °C for 3 min and then chilled to 4 °C. Next, 1x first-strand buffer (50 mM Tris-HCl pH 8.3, 75 mM KCl and 3 mM MgCl_2_), 5 mM dithiothreitol, 10 mM additional MgCl_2_ (all Thermo Fisher Scientific), 1 M betaine (Sigma-Aldrich), 0.6 μM adapter sequence-containing template switching oligonucleotide (5′-AAGCAGTGGTATCAACGCAGAGTACATrGrG+G-3′ where rG = riboguanosine and +G = locked nucleic acid modified guanosine, Eurogentec), 15 U RNaseOUT and 150 U SuperScript II (both Thermo Fisher Scientific) were added to a final volume of 15 μl. Final RT concentrations are shown. RT was performed at 42 °C for 90 min and 70 °C for 15 min. cDNA were stored at −20 °C.

Universal RT for optimal yield was performed using total RNA as published^15^ with minor changes. Briefly, 1.7 μM oligo-dT_15_, 1.7 μM random hexamers, 0.3 mM dNTPs (all Sigma-Aldrich) and 100 pg total RNA were incubated in 6.5 μl at 65 °C for 5 min and then chilled to 4 °C. Next, 1x first-strand buffer, 5 mM dithiothreitol, 15 U RNaseOUT, and 75 U SuperScript III enzyme (all Thermo Fisher Scientific) were added to a final volume of 15 μl. Final RT concentrations are shown. RT was performed at 25 °C for 5 min, 50 °C for 60 min, 55 °C for 15 min and 70 °C for 15 min. cDNA were stored at −20 °C. The protocol for universal RT using SuperScript II can be found in Supplementary Method.

### Preamplification

For adapter-based preamplification, 4.5 μl of cDNA generated from either total RNA samples or single cells was preamplified in a volume of 30 μl containing 1x KAPA HiFi HotStart Ready Mix (KAPA Biosystems) and 0.1 μM primer (5′-AAGCAGTGGTATCAACGCAGAGT-3′; Sigma-Aldrich). Adapter-based preamplification was performed at 98 °C for 3 min followed by 24 cycles of amplification at 98 °C for 20 s, 67 °C for 15 s, and 72 °C for 6 min and a final additional incubation at 72 °C for 5 min. Samples were moved from 72 °C directly to dry ice and stored at −20 °C. Preamplified cDNA was diluted 1:20 with 1x TE buffer (pH 8.0, Thermo Fisher Scientific).

Target-specific preamplification was performed on cDNA generated from total RNA samples as described^15^. Briefly, 4.5 μl of cDNA was preamplified in a volume of 30 μl containing 1x TATAA SYBR Grandmaster Mix (TATAA Biocenter) and 96 primer pairs, 40 nM of each primer. Primer sequences are shown in Supplementary Table 1. Multiplex preamplification was performed at 95 °C for 3 min followed by 20 cycles of amplification at 95 °C for 20 s, 60 °C for 3 min, and 72 °C for 20 s and a final additional incubation at 72 °C for 10 min. Samples were moved from 72 °C directly to dry ice and stored at -20 °C. Preamplified cDNA were diluted 1:20 with 1x TE buffer (pH 8.0).

Global and target-specific preamplification were monitored in real-time using SYBR Green I detection chemistry on a CFX384 Touch Real-Time PCR Detection System (Bio-Rad Laboratories) using cDNA generated by full-length and universal RT, respectively. RT was performed with 100 pg total RNA and cDNA corresponding to 30 pg total RNA was used for real-time monitoring of preamplification. Both preamplification approaches were performed as described above, except for the global preamplification which was adapted to real-time monitoring by adding 0.1x (final concentration) SYBR Green I (Sigma-Aldrich) to the reaction. Preamplification was monitored for 35 cycles followed by melting curve analysis ranging from 65 °C to 98 °C, 5 s per 0.5 °C increment.

### Targeted mRNA analysis using quantitative real-time PCR

Quantitative real-time PCR was performed in the CFX384 Touch Real-Time PCR Detection System using 6 μl reactions containing 1x TATAA SYBR Grandmaster Mix, 400 nM of each primer and 2 μl preamplified and diluted cDNA generated from single cells. The same primers were used as in multiplex PCR (see Supplementary Table S1). Quantitative real-time PCR was performed at 95 °C for 1 min followed by 35 cycles of amplification at 95 °C for 5 s, 60 °C for 30 s, and 72 °C for 10 s followed by a melting curve ranging from 65 °C to 95 °C, 5 s per 0.5 °C increment. All assays have been validated for specificity using gel electrophoresis. Cycle of quantification (Cq) values were determined by the second derivative maximum method.

High-throughput qPCR was performed on a BioMark system using the 96.96 Dynamic Array Chip for Gene Expression (Fluidigm). Each 5 μl sample contained 2 μl preamplified and diluted cDNA generated from total RNA samples, 2.5 μl 2x SsoFast EvaGreen SuperMix (Bio-Rad Laboratories), 0.25 μl DNA Binding Dye Sample Loading Reagent (Fluidigm), 0.01 μl 100x ROX (Thermo Fisher Scientific). The 5 μl assay reaction mixes contained 2.5 μl Assay Loading Reagent (Fluidigm) and 2.5 μl of mixed reverse and forward primers with a final concentration of 2.5 μM. The dynamic array was primed and loaded as recommended by the manufacturer using the IFC controller HX. The system was run at 70 °C for 40 min for thermal mixing and 60 °C for 30 s followed by 95 °C for 60 s and 40 cycles of amplification at 96 °C for 5 s and 60 °C for 20 s. Melting curve was performed ranging from 60 °C to 95 °C, 1 s per 0.5 °C increment. Data were analysed using the Fluidigm Real-Time PCR Analysis software (Fluidigm) applying the linear derivative baseline subtraction method and a user-defined global threshold to obtain Cq-values.

All qPCR experiments were conducted according to the Minimum Information for Publication of Quantitative Real-Time PCR Experiments guidelines^35^. Detailed step-by-step qPCR data analyses, including principal component analysis, were performed as described using GenEx version 6 (MultiD)^28^. All qPCR and high-throughput qPCR data are reported as relative quantities in log_2_-scale, except coefficient of variation which was calculated in linear scale. Statistical analyses and plots were done in GraphPad Prism 7 (GraphPad Software).

## Additional Information

**How to cite this article:** Kroneis, T. *et al*. Global preamplification simplifies targeted mRNA quantification. *Sci. Rep.* **7**, 45219; doi: 10.1038/srep45219 (2017).

**Publisher's note:** Springer Nature remains neutral with regard to jurisdictional claims in published maps and institutional affiliations.

## Supplementary Material

Supplementary Information
