# Supplementary material for: Global preamplification simplifies targeted mRNA quantification
Source: Sci Rep. 2017 Mar 23;7:45219. doi: 10.1038/srep45219 (PMC5362892; doi:10.1038/srep45219)
Supplement: Supplementary Information [file srep45219-s1.pdf]

Global preamplification simplifies targeted mRNA quantification

Thomas Kroneis, Emma Jonasson, Daniel Andersson, Soheila Dolatabadi, and Anders Ståhlberg

Supplementary Method

We performed universal reverse transcription (RT) using SuperScript II according to the manufacturer's recommendations. Briefly, 1.5 µM oligo-dT_{15}, 1.5 µM random hexamers, 0.5 mM dNTPs (all Sigma-Aldrich) and 100 pg total RNA were incubated in 6.5 µL at 65°C for 5 min and then chilled to 4°C. Next, 1x first-strand buffer, 10 mM DTT, 30 U RNaseOUT, and 38 U SuperScript II enzyme (all Thermo Fisher Scientific) were added to a final volume of 15 µL. Final RT concentrations are shown. RT was performed at 25°C for 10 min, 42°C for 90 min, and 70°C for 15 min. cDNA was stored at -20°C.

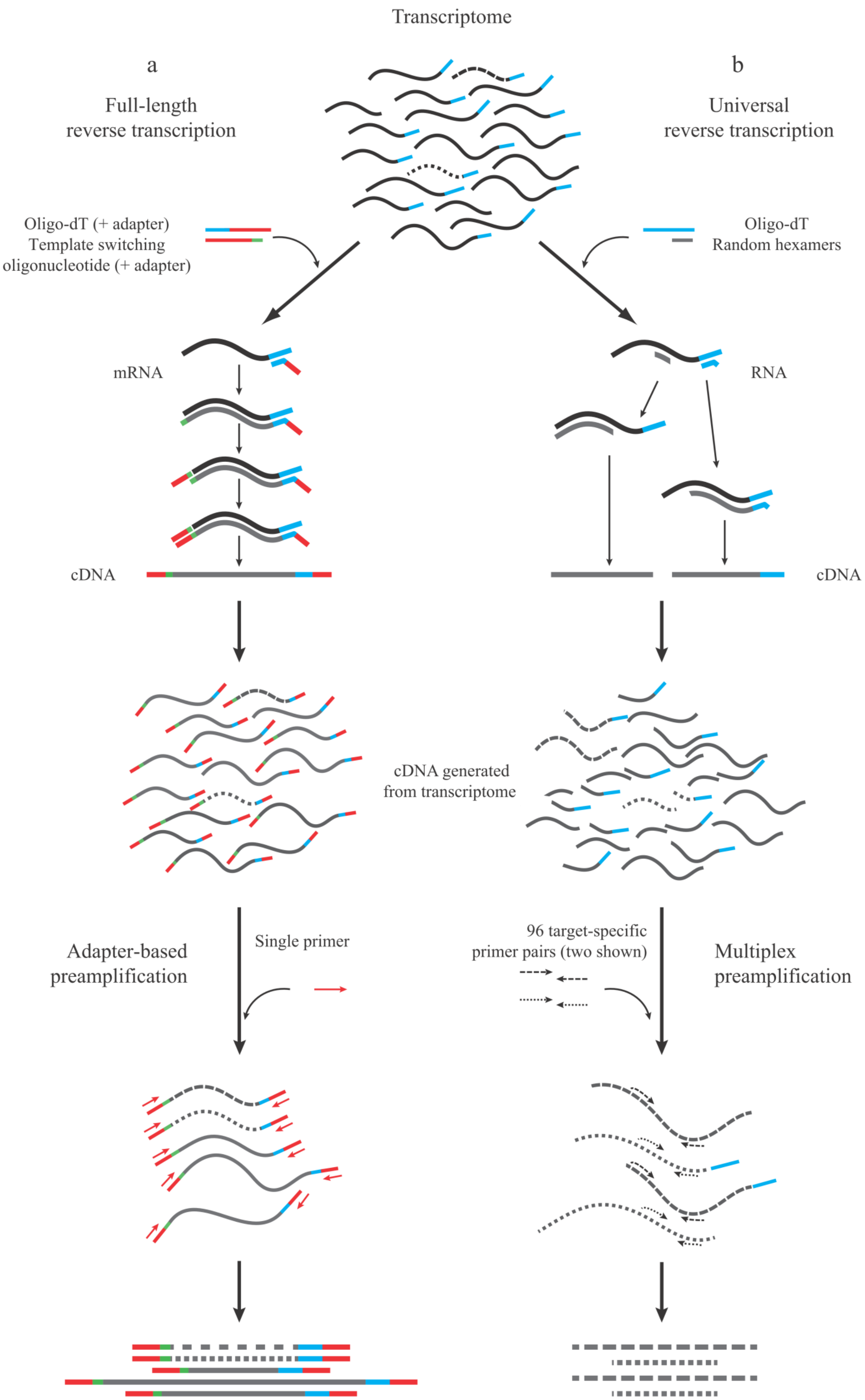

Transcriptome
a
Full-length
reverse transcription
b
Universal
reverse transcription
Oligo-dT (+ adapter)
Template switching
oligonucleotide (+ adapter)
Oligo-dT
Random hexamers
mRNA
RNA
cDNA
cDNA
cDNA generated
from transcriptome
Adapter-based
preamplification
Single primer
96 target-specific
primer pairs (two shown)
Multiplex
preamplification

Supplementary Figure S1. Detailed outline of global and target-specific preamplification. (a) In global preamplification, mRNA is full-length reverse transcribed. First, the mRNA poly-A tail is primed with a primer containing both an oligo-dT sequence (blue) and an adapter sequence (red). Reverse transcriptase generates full-length cDNA (grey) and adds 3-5 extra residues of cytosine (green) at the 3’ cDNA end. With the help of a template switching oligonucleotide (red) containing three guanine residues (green), reverse transcriptase can synthesise an adapter sequence attached to the 3’ cDNA end (full-length reverse transcription). A single adapter primer is used to preamplify the whole transcriptome (adapter-based preamplification). (b) In target-specific preamplification, a blend of random hexamers (grey) and oligo-dT (blue) primes total RNA, generating cDNA (grey) (universal reverse transcription). A pool of all target primers is used to preamplify the sequences of interest (multiplex preamplification).

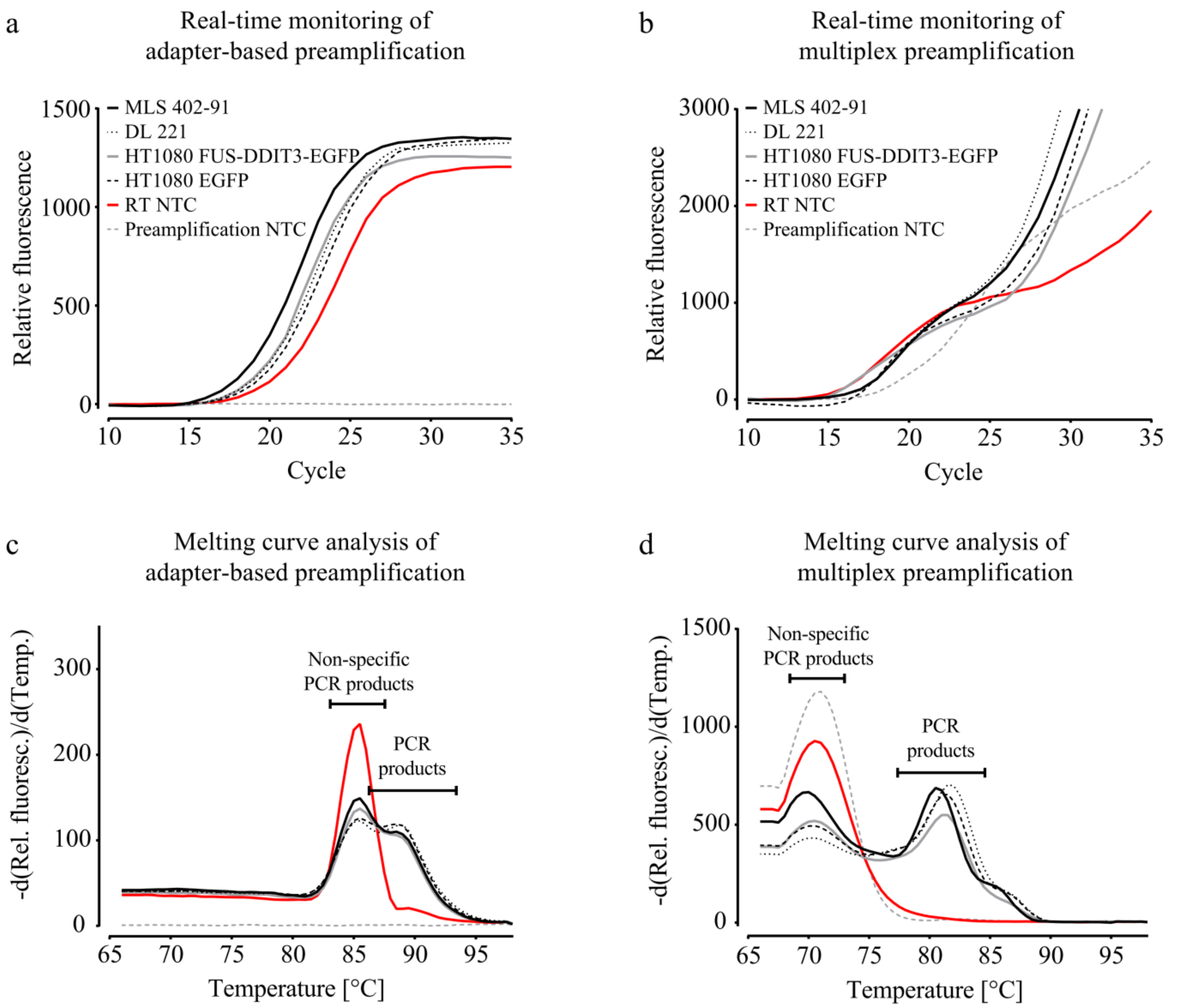

Supplementary Figure S2. Real-time monitoring of preamplification using RNA from different cell lines. (a) Response curve for real-time monitoring of adapter-based preamplification using SYBR Green I detection chemistry following full-length reverse transcription (RT). (b) Response curve for real-time monitoring of multiplex preamplification using SYBR Green I detection chemistry following universal RT. cDNA corresponding to 30 pg total RNA isolated from respective cell line was used in each preamplification reaction. Total RNA from cell lines MLS 402-91, DL 221, HT1080 FUS-DDIT3-EGFP, and HT1080 EGFP was used. Melting curves for adaptor-based and multiplex preamplification are shown in (c) and (d), respectively. RT no-template controls (NTCs) and preamplification NTCs were included as references to distinguish specific from non-specific PCR products and to

determine in what reactions these products were generated. –d(Rel. fluoresc.)/d(Temp.), –d(Relative fluorescence)/d(Temperature).

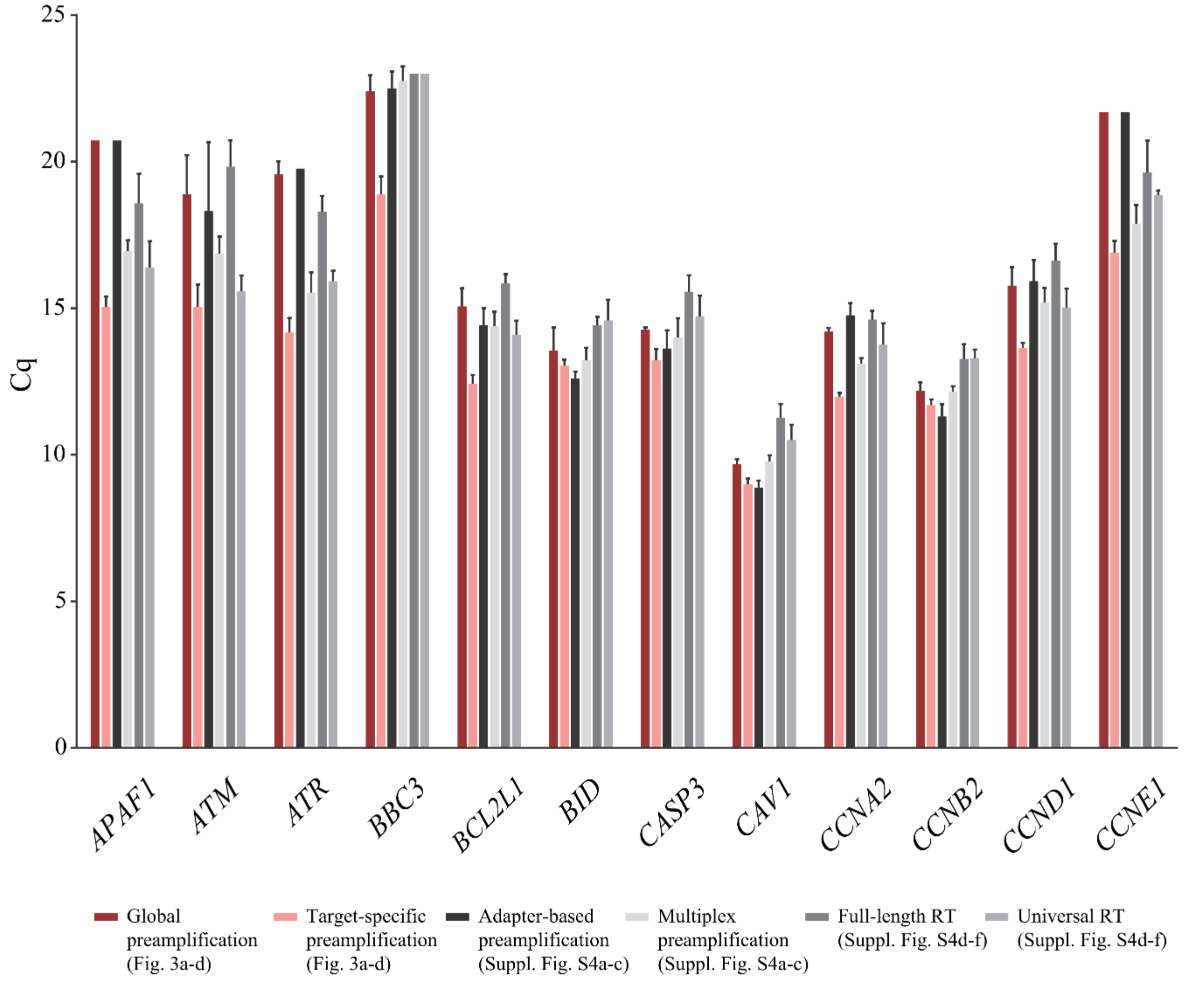

Supplementary Figure S3. Summary of all gene expression data. Missing data were assigned a cycle of quantification (Cq) value equal to the highest detected Cq-value plus 1 for respective gene. Mean ± SD is shown, n = 3-5. RT, reverse transcription.

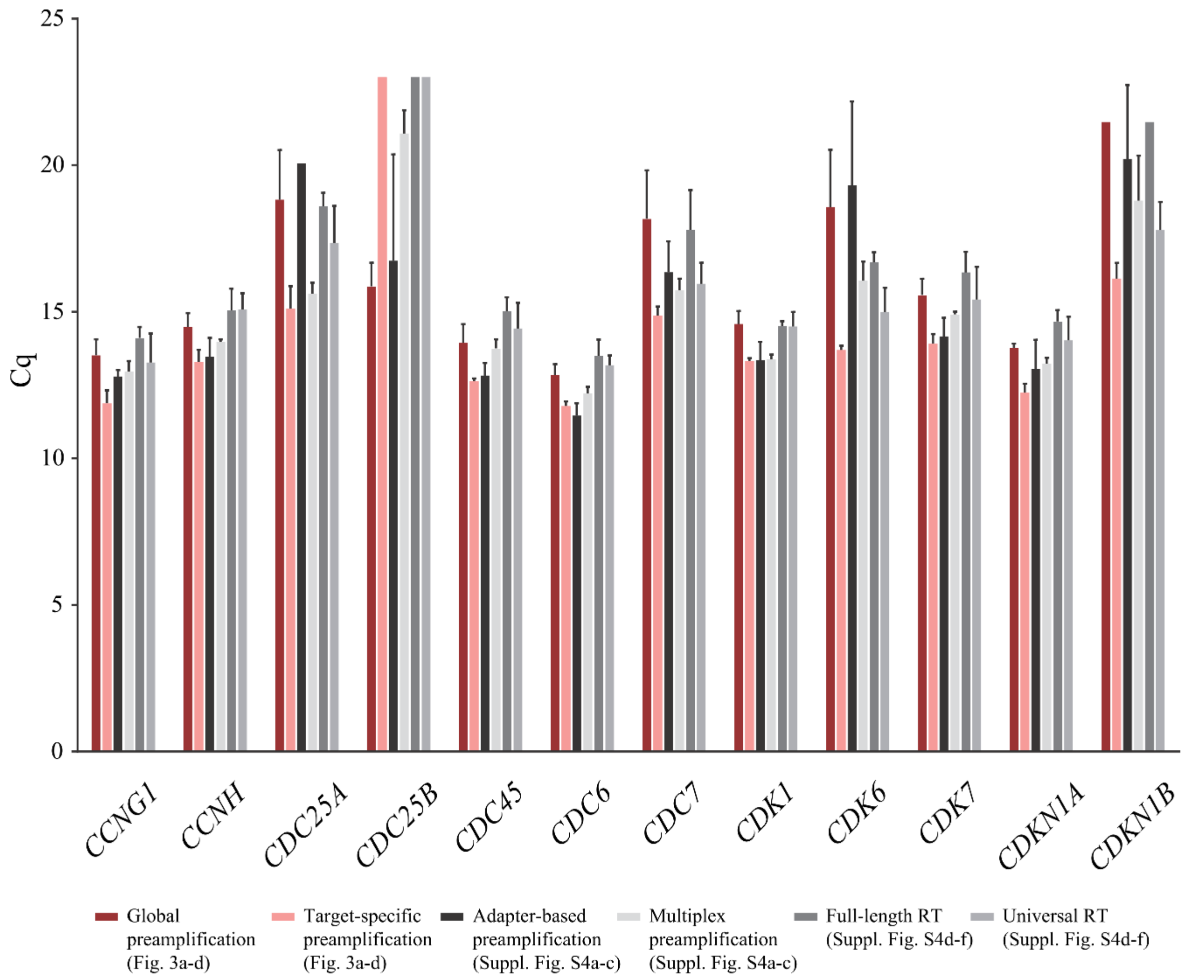

Supplementary Figure S3 continued

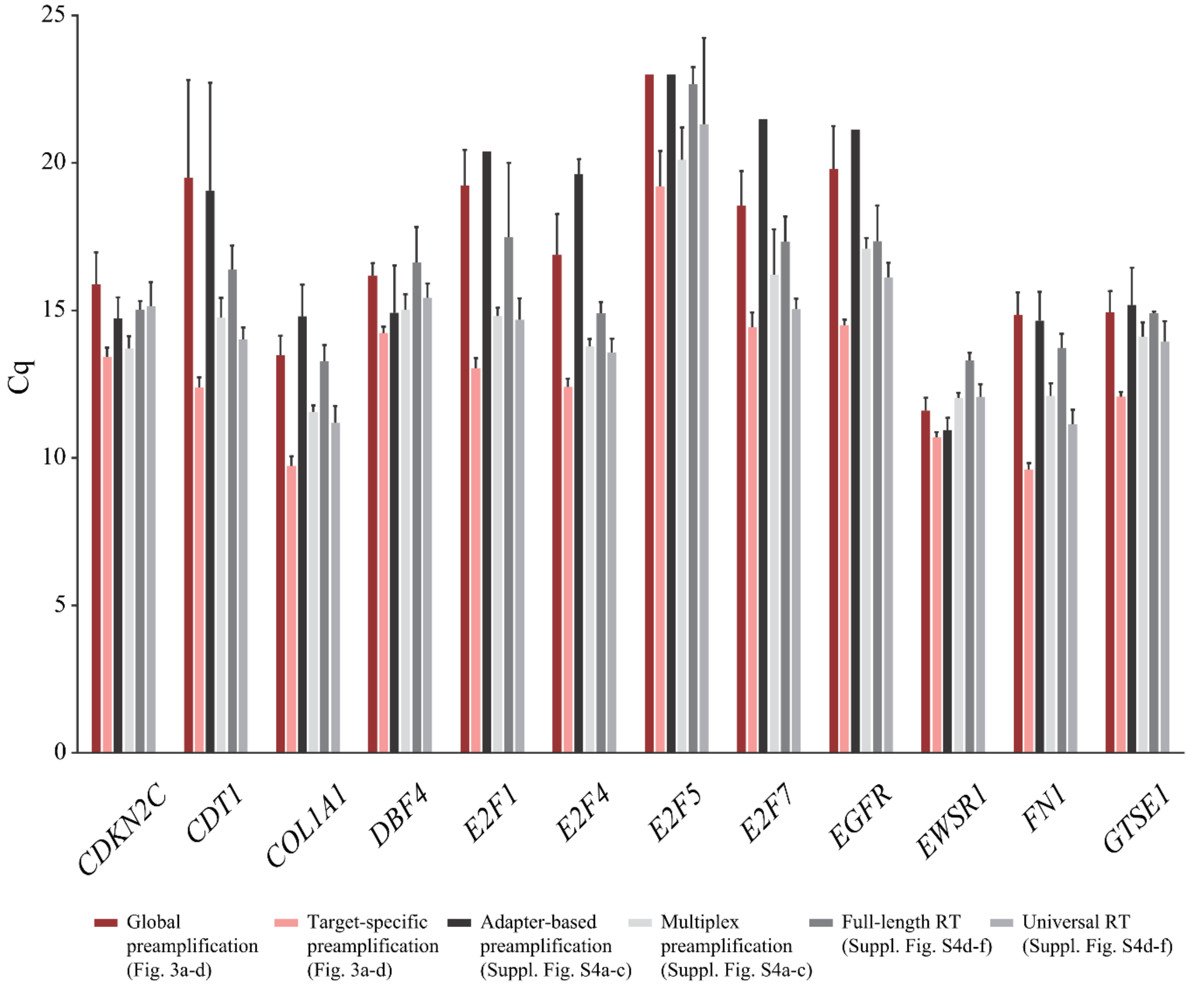

Supplementary Figure S3 continued

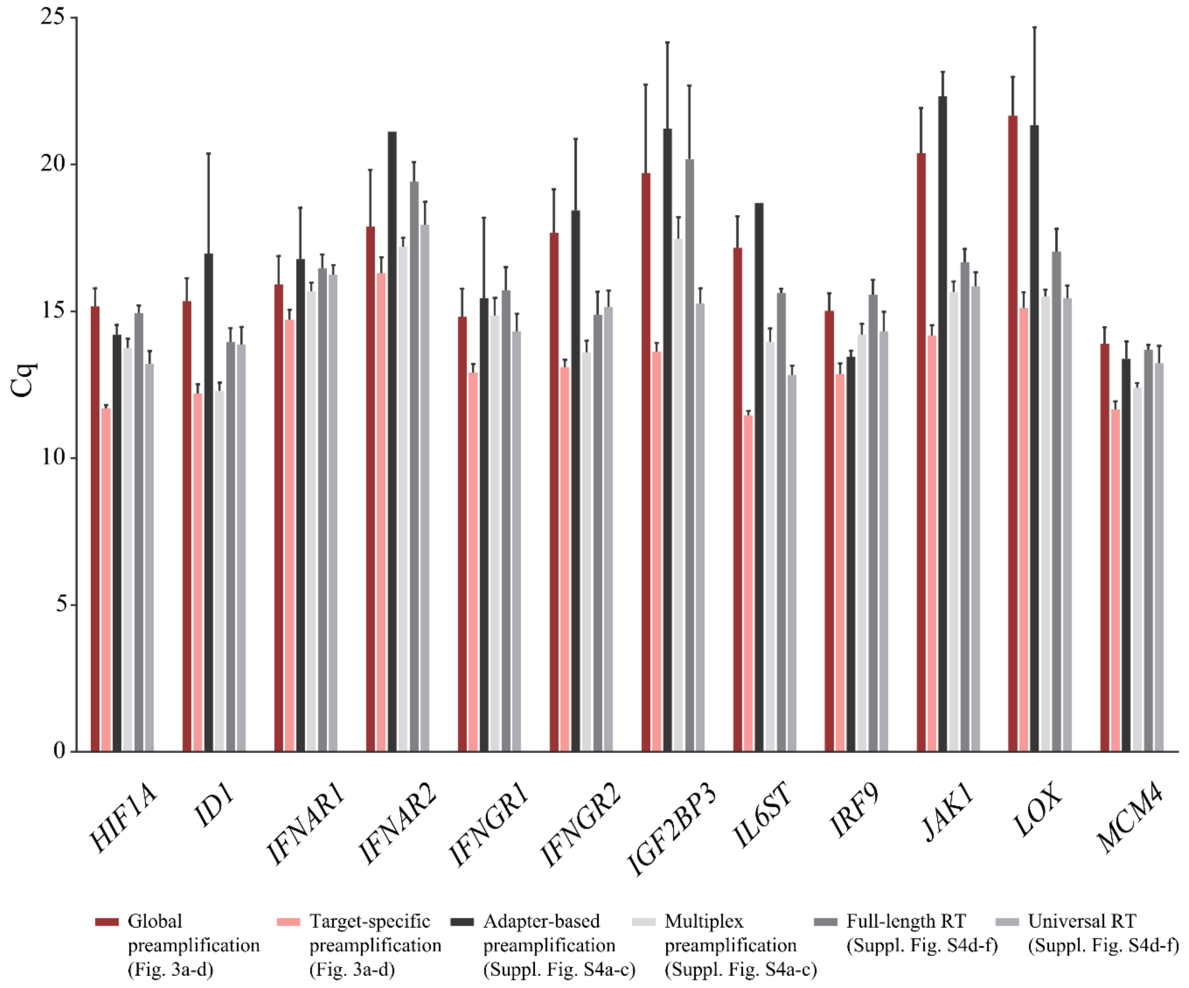

Supplementary Figure S3 continued

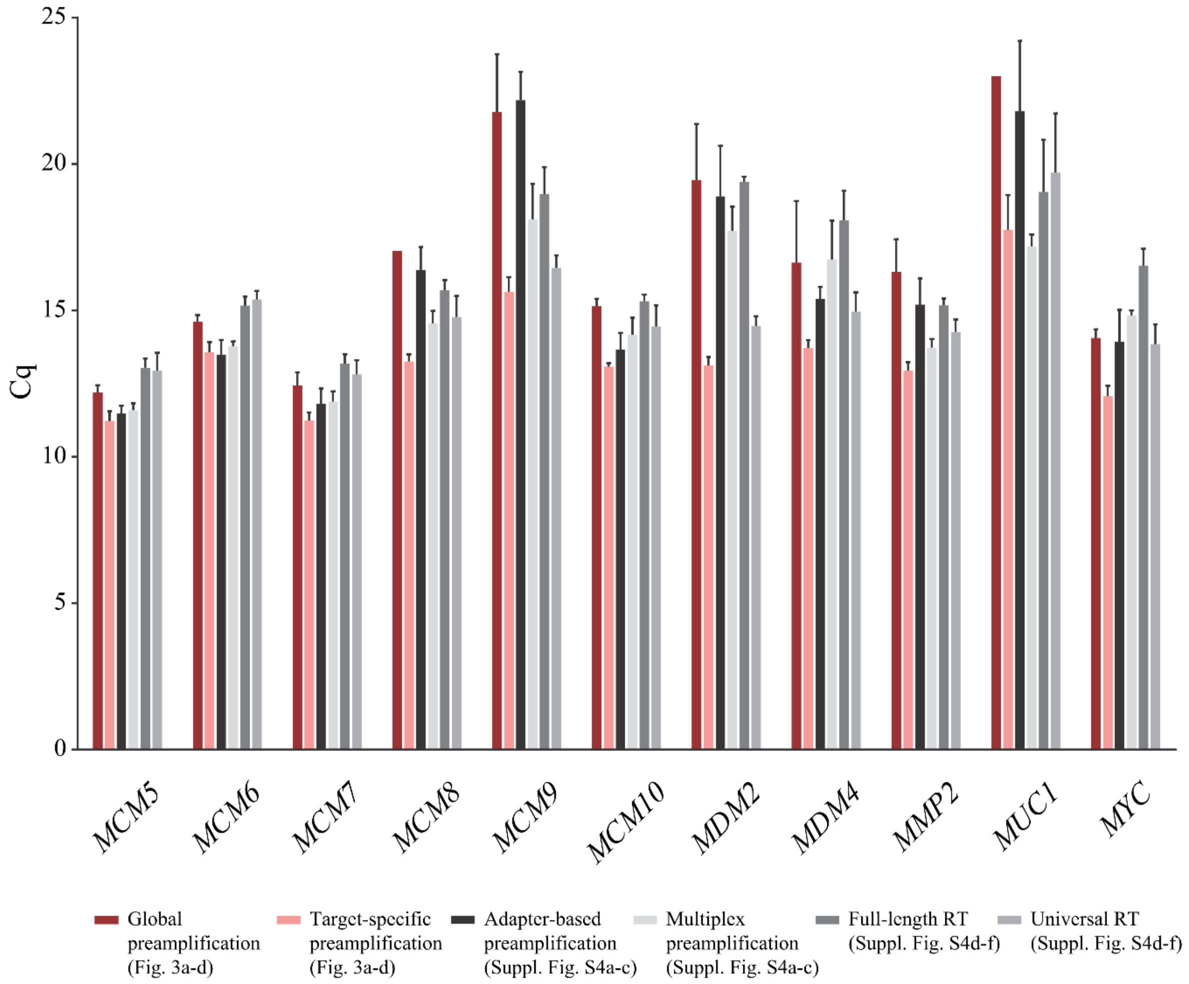

Supplementary Figure S3 continued

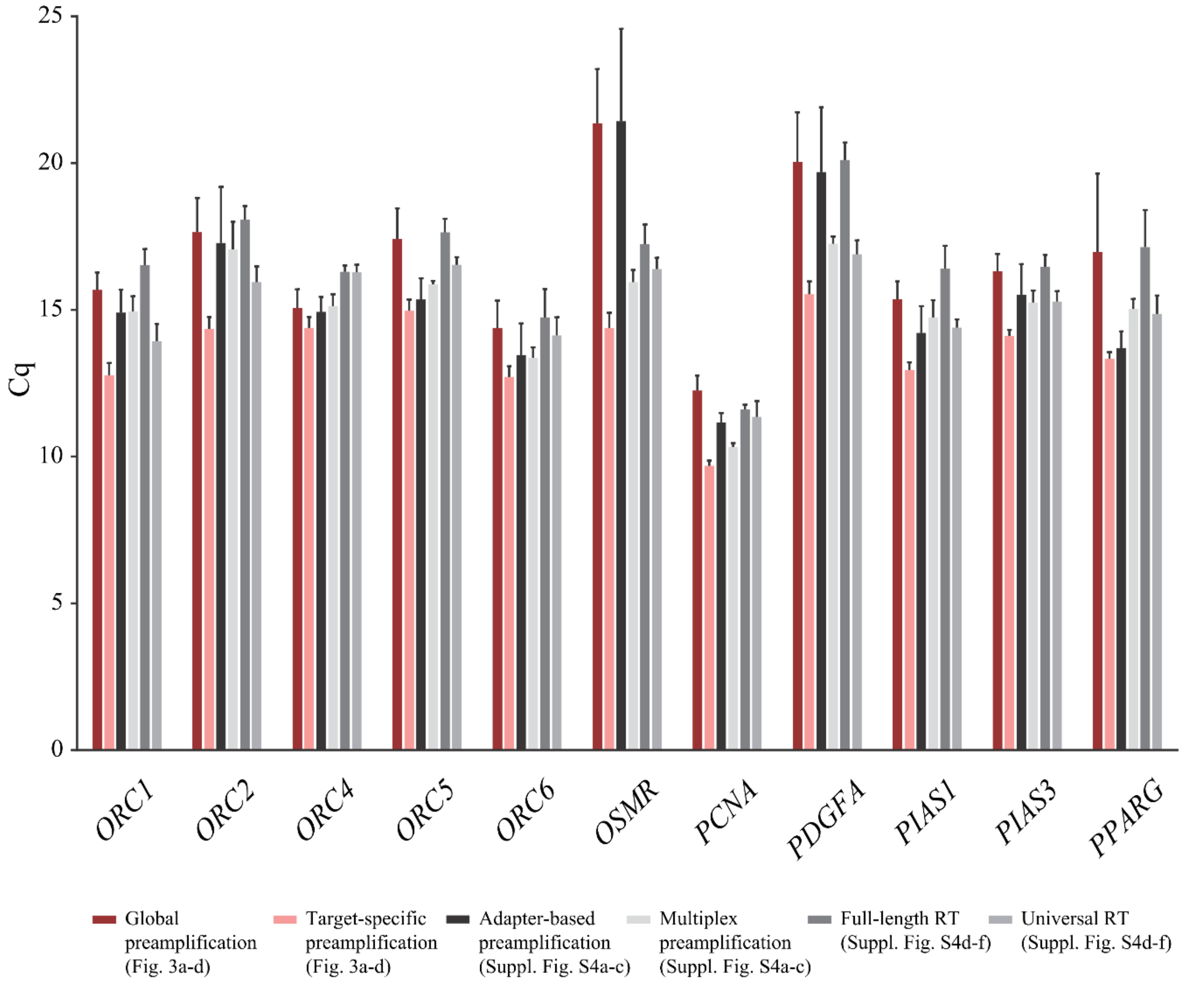

Supplementary Figure S3 continued

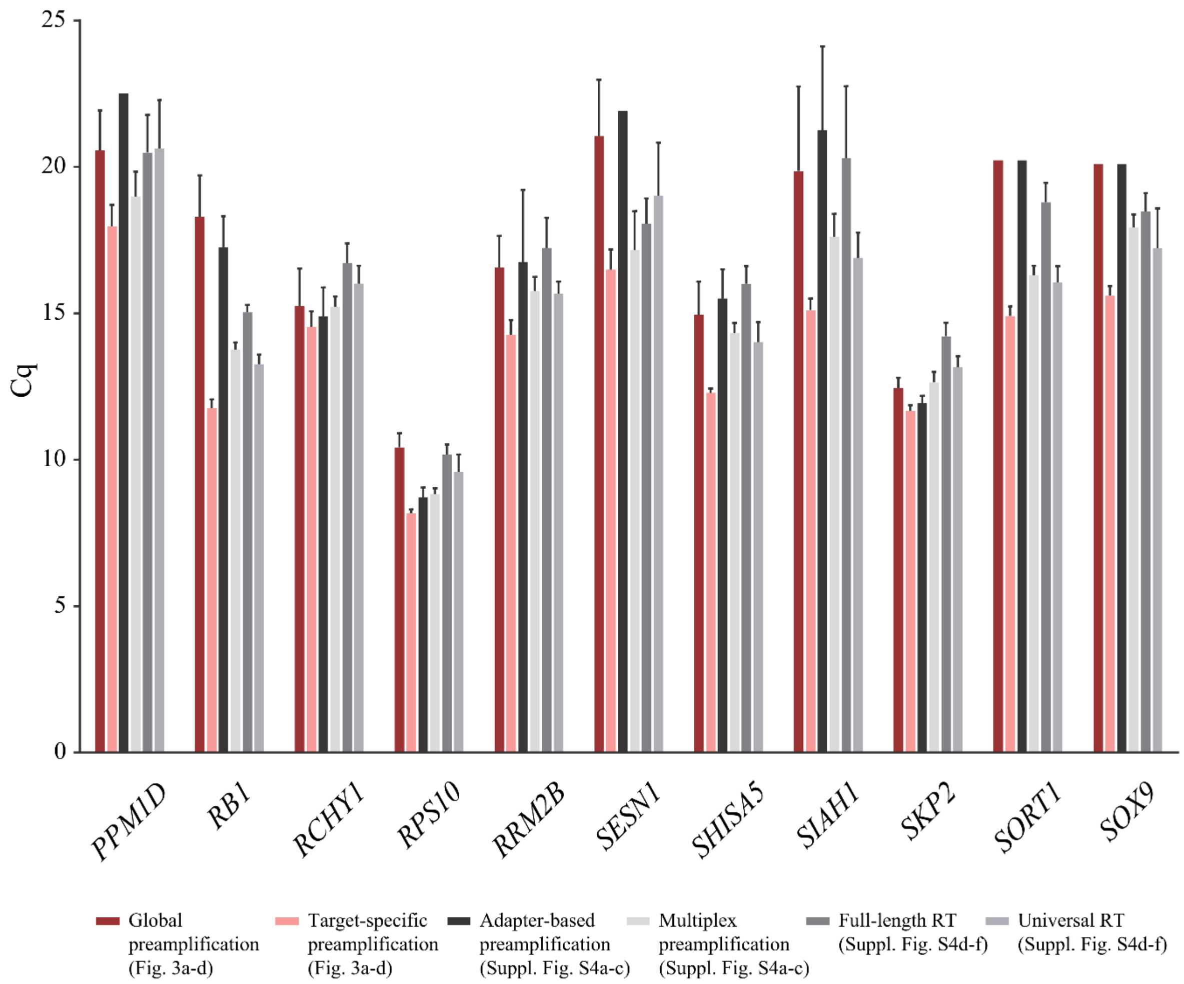

Supplementary Figure S3 continued

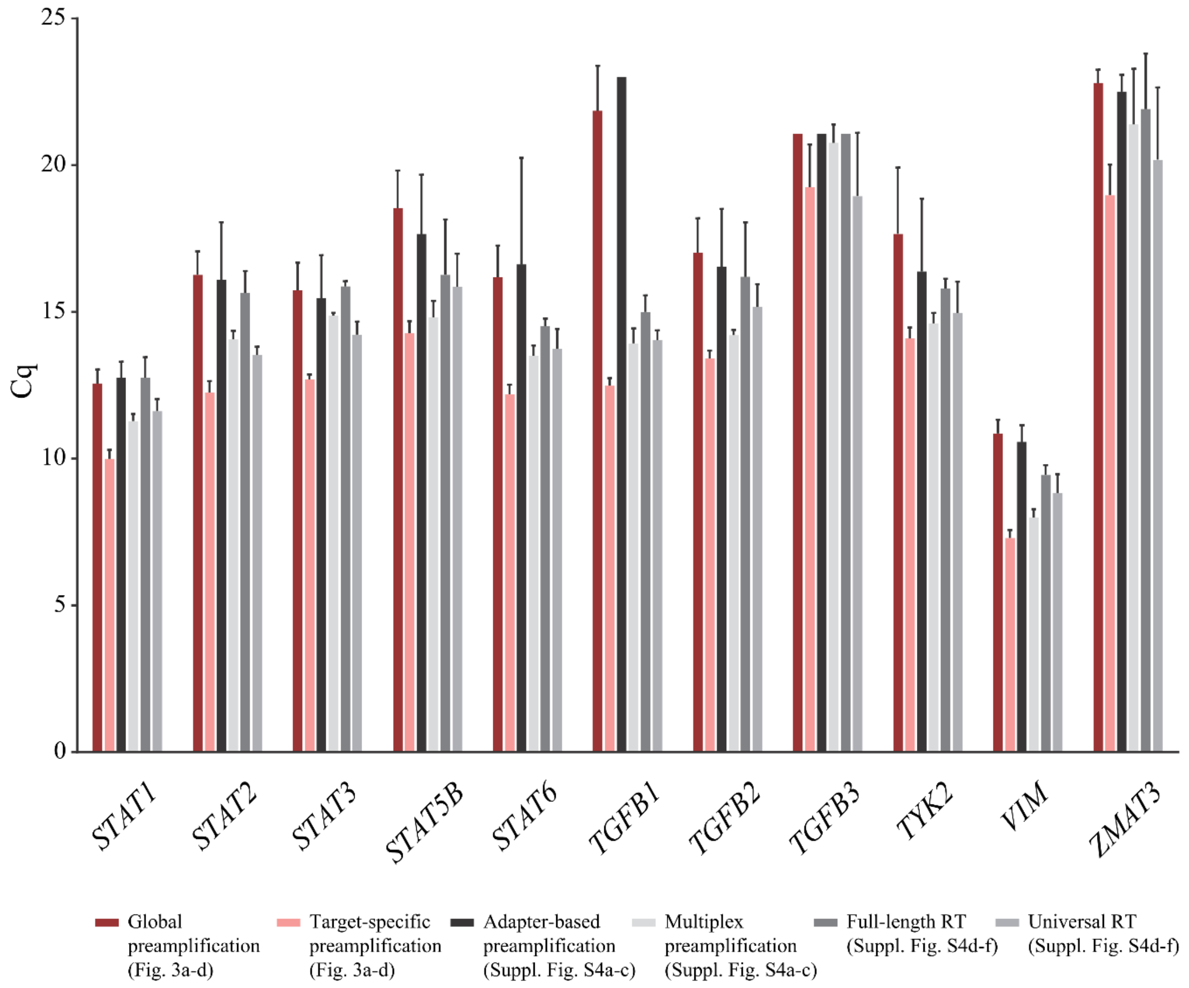

Supplementary Figure S3 continued

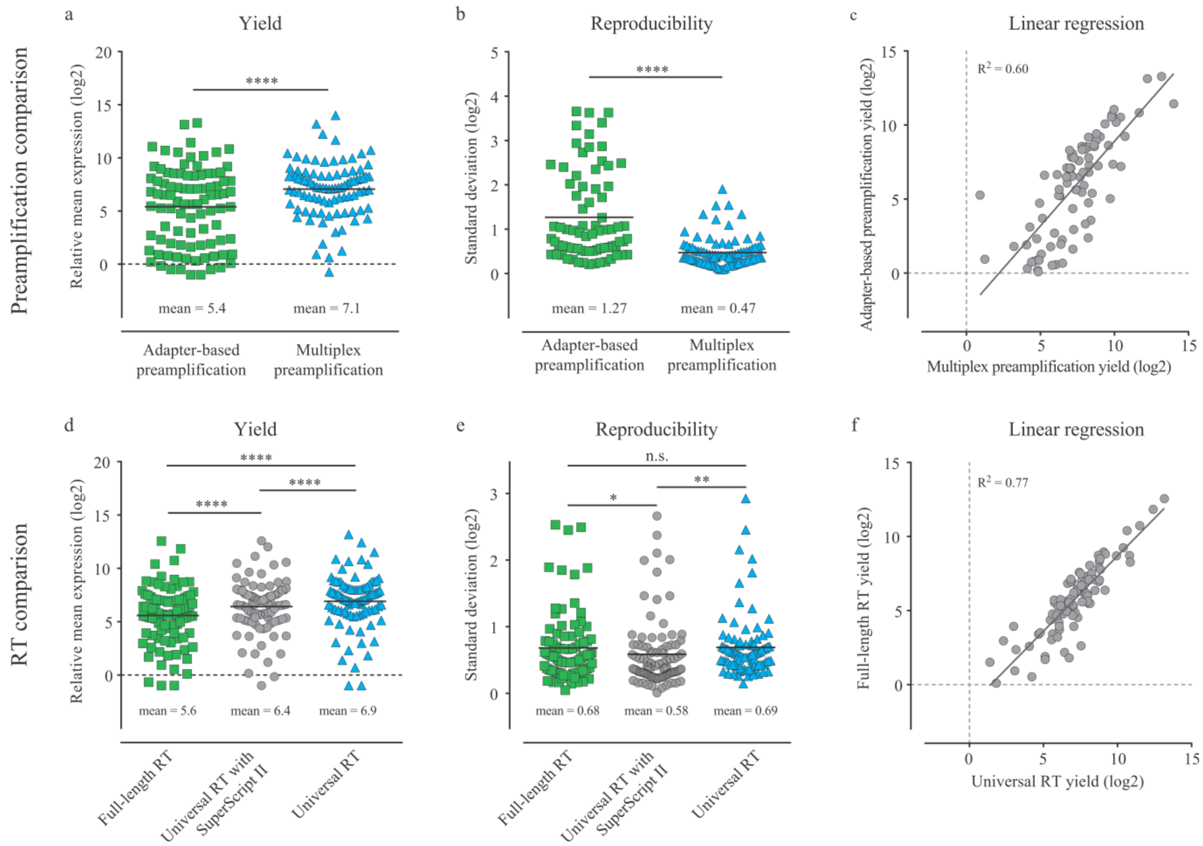

Supplementary Figure S4. Comparison between individual experimental steps in global and target-specific preamplification. (a) Relative yield and (b) reproducibility of adapter-based and multiplex preamplification using cDNA corresponding to 30 pg total RNA generated using full-length reverse transcription (RT). Each assay is indicated by squares (adapter-based, n = 4) and triangles (multiplex, n = 4). The horizontal solid bars indicate mean values and the horizontal dashed line indicates one molecule. **** indicates $p < 0.0001$ using Wilcoxon matched-pairs signed rank test, n = 92. (c) Linear regression comparing the yield of adapter-based and multiplex preamplification.(d) Relative yield and (e) reproducibility of full-length RT (squares, n = 3), universal RT with SuperScript II (circles, n = 3), and universal RT (triangles, n = 3) using 100 pg total RNA. Each square (full-length RT, n = 3), circle (universal RT using SuperScript II, n = 3) and triangle (universal RT, n = 3) represents one assay. The horizontal solid bars indicate mean values and the horizontal dashed line indicates one molecule. ****, **, * indicate $p < 0.0001$, $p < 0.01$ and $p < 0.05$, respectively, using

Wilcoxon matched-pairs signed rank test, n = 92. (f) Linear regression comparing the yield of full-length and universal RT.

a b

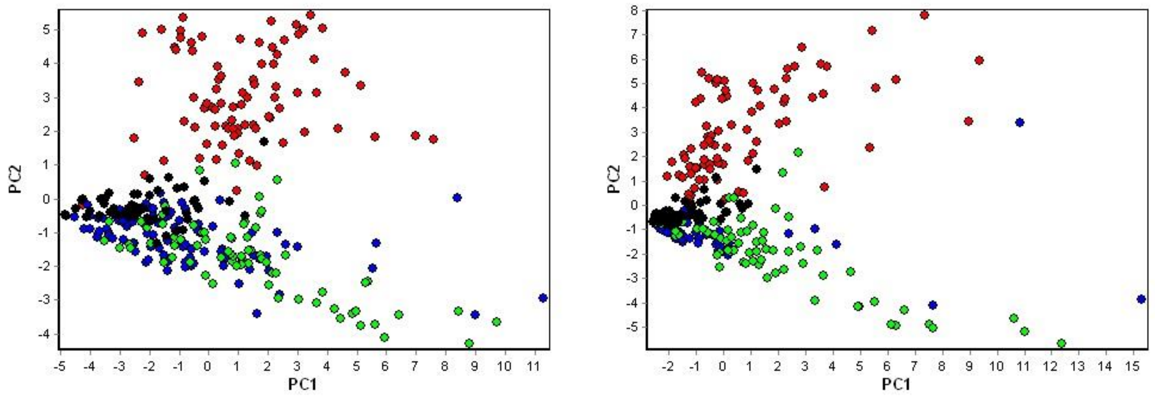

Supplementary Figure S5. Effect of reduced sensitivity on single-cell gene expression profiling. (a) Principal component analysis (PCA) of single cells based on target-specific preamplification using a published data set [28]. (b) PCA based on same data set as in (a). Here, all relative copy numbers have been divided by 9.3 and resulting values < 1 were removed to mimic the reduced yield obtained by global preamplification. Similar PCA is also generated with even higher cut-off (data not shown). Data points represent individual astrocytes derived from mouse brains before (day 0, black), and after (day 3, blue, day 7, lime, and day 14, red) ischemia.

Supplementary Table 1: Assay information

Gene	Accession number	Forward sequence (5'-3')	Reverse sequence (5'-3')	Intron spanning
APAF1	NM_013229.2	ATGGAACAGTGAAGGTATGGAA	AGCAGAGGTAGATGAAAACTTGGT	Yes
ATM	NM_000051.3	CCGTCAGCAAAGAAGTAGAAGGA	GCTAAGTGTGGGATAGAGCGA	Yes
ATR	NM_001184.3	GGATGCCACTGCTTGTTATGA	TCCACTCGGACCTGTTAGC	Yes
BBC3	NM_001127240.2	TACGAGCGGCGGAGACAAG	CTGGGTAAGGGCAGGAGTC	Yes
BCL2L1	NM_138578.1	TGCGTGGAAAGCGTAGACAA	ACAAAAGTATCCCAGCCGCC	Yes
BID	NM_197966.2	GCCAGAAATGGGATGGACTGA	TCTCTAGGAACGCTGTTGACAT	Yes
CASP3	NM_032991.2	GGCGTGTCATAAAATACCAGTG	GCGTCAAAGGAAAAGGACTCAAA	Yes
CAV1	NM_001753.4	GCAACATCTACAAGCCCAACA	GCCTTCCAAATGCCGTCAAAA	Yes
CCNA2	NM_001237.4	AAGACGAGACGGGTTGC	GGCTGTTTACTGTTTGCTTTCC	Yes
CCNB2	NM_004701.3	CGACCCTTGCCACTACACTT	TGACTTCCAATACTTCATTCTCTG	Yes
CCND1	NM_053056.2	AGAGGCGGAGGAGAACAAA	TGAGGCGGTAGTAGGACAGG	Yes
CCND3	NM_001760.3	CTGTTCGCTGCCCGAGTATG	GCATGTGCGGCTTGATCTC	No
CCNE1	NM_001238.3	CCGGTATATGGCGACACAAG	TACGCAAACTGGTGCAACTT	Yes
CCNG1	NM_004060.3	CCTTGGGTGTGTTGGACTGA	GCTATTTCTCCTTTCAAGTGGCA	Yes
CCNH	NM_001239.3	TGAGGAGGAAGAATGGACTGA	GTTACTGTGAAAGGGAAAGAAAACA	Yes
CDC25A	NM_001789.2	GTCGCCTGTCACCAACCT	CGGAGGAGCCCATTCTCT	Yes
CDC25B	NM_021873.3	GGCGGGCACATCAAGACTG	GGGTAGTAGAGGCTGGGGTA	Yes
CDC45	NM_001178010.2	CTTTGAGTATGACCTCCGCCT	AACTTCTGCTTCACCTGCTTCA	Yes
CDC6	NM_001254.3	ACTTGGTGCTGATTGGTATTGC	TGCGAACATCTCCTGAAACA	Yes
CDC7	NM_003503.3	ACACCAGGATTCAGAGCACC	TCGTCCACTAAGCAAAGAAAGAA	Yes
CDK1	NM_001786.4	AGAAAGTGAAGAGGAAGGGGTTC	AGCACATCCTGAAGACTGACTA	Yes
CDK6	NM_001259.6	CGTGGTCAGGTTGTTTGATGT	CGGTGTGAATGAAGAAAGTCC	Yes
CDK7	NM_001799.3	GCCAGAGATAAGAACACCAACC	TCCAAAAGCATCAAGGAGACCA	Yes
CDKN1A	NM_000389.4	TTAGCAGCGGAACAAGGAGT	GCCGAGAGAAAACAGTCCAG	No
CDKN1B	NM_004064.4	TCTGAGGACACGCATTTGGT	CTTAATTCGAGCTGTTTACGTTTGA	Yes
CDKN2C	NM_001262.2	AGTTCCTGGTGAAGCACACGG	CCTCATTCCTCCCATAGAGCCT	No
CDT1	NM_030928.3	GACTCGTGCTGCCCTACAA	ACTCCTCAAAACGCCTACG	Yes
COL1A1	NM_000088.3	CGGAGCAGACGGGAGTTTC	TGTACGCAGGTGATTGGTGG	Yes
DBF4	NM_006716.3	GCCAGAAAAATCCAAATGTAAGCC	TTCAACTCGCCCTCCCAGAT	Yes
E2F1	NM_005225.2	TGCCAAGAAGTCCAAGAACCA	GTCAACCCCTCAAGCCGTC	Yes
E2F4	NM_001950.3	CGGACCCAACCCTTCTACCT	GGGGCAAACACTTCTGAGGA	Yes
E2F5	NM_001951.3	TGATACTTTGGCTGTGAGGCA	CAGCACCTACACCTTTCCAC	Yes
E2F7	NM_203394.2	CCTTTAGCCCACCCAGTATTT	ATCCCTCTCTGACCCTGACC	No
EGFR	NM_005228.3	AGAGCGACTGCCTGGTCT	TACGGGGACACTTCTTCACG	Yes
EWSR1	NM_013986.3	ATACTACTCCAACTGCCCCC	TCCATCCTGCGGTCTTGTA	Yes
FN1	NM_212482.1	CTGAGGGCAGAAGAGACAACA	CCACGACCATTCCCAACACA	Yes
GTSE1	NM_016426.6	TGCGGAGAAGCCCAAGAAAGAG	TGCGAGATTGCTGGTAGAGCC	Yes
HIF1A	NM_001530.3	CGATTTTGGCAGCAACGACACA	CGTTTCAGCGGTGGGTAATGGA	Yes
ID1	NM_002165.3	CTGAGGGAGAACAAGACCGAT	CCCCCTAAAGTCTCTGGTGA	No
IFNAR1	NM_000629.2	GTGAGAAAACAAAACCAGGAAATAC	TGACAAACGGGAGAGCAAAT	Yes
IFNAR2	NM_207585.2	GGATTCAGCGGGAACACA	CCTTTTATTTCGGGTTTATGCTTCT	Yes
IFNGR1	NM_000416.2	TCCAGTTGTTGCTGCTTTACT	AACGGCTCTTCACAGACCAC	Yes

IFNGR2	NM_005534.3	TTTTCGTTGCTGTCGGTGC	TGGGCTGAGTTGGGTCTTTT	Yes
IGF2BP3	NM_006547.2	TCCCAAAAAGGCAAAGGATTCG	GCTCTCCACCACTCCATACTG	Yes
IL6ST	NM_002184.3	GAGGTGTGAGTGGGATGGTG	GCGGATTGGGCTTCACTTTA	Yes
IRF9	NM_006084.4	CCATCAAAGCGACAGCACAG	GCCCCCTCCTCCTCATTATT	Yes
JAK1	NM_002227.3	CTGGAGTATCTGTTTGCTCAGG	GCTCGGTCTTGGGGTCTC	Yes
LOX	NM_002317.5	CCAGTACAGCATACAGGGCA	TGGCATCAAGCAGGTCATAG	Yes
MCM4	NM_005914.3	GCCAAACGCCTCCATCG	GGCACTCATCCCCGTAGTAAG	Yes
MCM5	NM_006739.3	ACTTCACCAAGCAGAAATACCCG	GGCAGAGGTCCCAGCAACAT	Yes
MCM6	NM_005915.5	AGCGGAACTTTTCTGTGCTT	CTTCTAAACTGCGGGGGATAC	Yes
MCM7	NM_005916.4	AACTGTGCGTGGAATCGTCA	GAGACTGGATCGGCTGGTAG	Yes
MCM8	NM_032485.5	TCTTCCCACAAAGTGTCCTGT	CCGACCTGCTTCTCTCTGAT	Yes
MCM9	NM_017696.2	AGGCTGGGGCATTAGTTCTT	ATGGTGGTCCTTGTGTTCAG	Yes
MCM10	NM_182751.2	CTTCTCTGGTCTGCGGCTC	AGGTTTTTCCACTATTCACACTCT	Yes
MDM2	NM_002392.5	ATCAGCAGGAATCATCGGAC	GTGGCGTTTTCTTTGTCGTT	Yes
MDM4	NM_002393.4	TGCCGCTTTTGAAGATTTTGC	GAGAGGGCTTGGGTCTTTCA	Yes
MMP2	NM_004530.5	GTCCGTGTGAAGTATGGGAAC	CCCTGGAAGCGGAATGGAAAC	Yes
MUC1	NM_002456.5	CTGGTCTGTGTTCTGGTTGC	CCACTGCTGGGTTTGTGTAA	Yes
MYC	NM_002467.4	GGAGGCTATTCTGCCCATTT	GGCTGCTGGTTTTCCACTAC	Yes
ORC1	NM_004153.3	CAAGCCTAGAACGCCACG	TACATGCACCCTCCGGTATG	Yes
ORC2	NM_006190.4	ACCTAGCGGTGACTGTATCTG	CTCCCACAAAGTGAACCTCCA	Yes
ORC4	NM_002552.4	ACACATGCTATTGATGCTTGCT	TGCTACACAGTTGGCTTGCT	Yes
ORC5	NM_002553.3	TGCCAGAATGCCCCACTT	TGATGTCTCTCTCCAAACAAGGA	Yes
ORC6	NM_014321.3	GAAGCCCCAGCAAAGGAAATG	AAATCCCAAAGCCGTCAAGT	Yes
OSMR	NM_003999.2	TGTCATCTGGGTGGGGAAT	CTCAGGGAACTTGGCATCGT	Yes
PCNA	NM_002592.2	GTGGAGAACTTGGAAATGGAA	ACCGTTGAAGAGAGTGGAGTG	Yes
PDGFA	NM_002607.5	CACCACCGCAGCGTCAA	CCGTGTCCTCTTCCCGATAA	Yes
PIAS1	NM_016166.1	ACCTGTCCTTCCCTATCTCCC	GGTGTTGTAATGCTGATTGTCTCC	Yes
PIAS3	NM_006099.3	GAGCCGACATCCAAGGTTTAG	CCAGAAAGTGAGAAGGGGTCC	Yes
PPARG	NM_138712.3	TACTCCACATTACGAAGACAT	CTCCATAGTGAAATCCAGAAG	Yes
PPM1D	NM_003620.3	AAGGGTTTCACCTCGTCCG	GCCATTCCGCCAGTTTCTTC	Yes
RB1	NM_000321.2	AAAGGACCGAGAAGGACCA	AAGGCTGAGGTTGCTTGTGT	Yes
RCHY1	NM_015436.3	ACTGTGGAATTTGTAGGATTGGT	ACATGAGCAACAACACGGGA	Yes
RPS10	NM_001014.4	AGCCGCAGAGATGTTGATG	CCTCGGGACTTGAGAGACTG	Yes
RRM2B	NM_015713.4	TGTGACTTTGCTTGCCTGATG	TGCCTGAAAAACCTTTGAGAATCC	Yes
SESN1	NM_014454.2	GGGAGTGAAGACGCACAGAT	GCCGCAGCCATTATTCCAA	Yes
SHISA5	NM_016479.4	GTGGTGAGGTGTGTATGGCTT	AGGTCGCTCCGAACCCTGA	Yes
SIAH1	NM_003031.3	CGCTCTCCGCCCACAGAAAT	GGACACTCAAAAAGACTCGCCA	Yes
SKP2	NM_005983.3	CCCCAGGAACTGCTCTCAAA	ACTCATCAGACGCTAGGCGA	Yes
SOCS2	NM_003877.4	CATGACCCTGCGGTGCCTT	AAGTTCCTTCTGGTGCCTCTTTT	Yes
SORT1	NM_002959.5	ATGGGAAGAAATCCACAAAGCAG	ATTCCAGAGCCCCAAGGTCAG	Yes
SOX9	NM_000346.3	GCTCTGGAGACTTCTGAACGA	CCGTTCTTCACCGACTTCCT	Yes
STAT1	NM_007315.3	GTTATGGGACCGCACCTTCA	CACCAACAGTCTCAACTTCACAG	Yes
STAT2	NM_005419.3	AGCACCAGGATGATGACAAGG	GGGGGATTCGGGGATAGAGG	Yes
STAT3	NM_139276.2	GCCAGAGAGCCAGGAGCATC	GGGACATCGGCAGGTCAAT	Yes
STAT5B	NM_012448.3	CTGCGAGTCTGCTACTGCTA	GAGTCAGGGTTCTGTGGGTA	Yes
STAT6	NM_001178078.1	GAACATCCAGCCATTCTCTGC	TTGGTCCCTTTCCACGGTCA	Yes
SUZ12	NM_015355.3	AGCCATCACCAAACTCAGAAA	GCTTTTTACCTGTGGGAACTTG	Yes

TGFB1	NM_000660.6	AACAATTCCTGGCGATACCTCA	AAGCCCTCAATTTCCCCTCC	Yes
TGFB2	NM_001135599.2	AAGACCCCACATCTCCTGCTAA	TCGTGTATCCATTTCCACCCT	Yes
TGFB3	NM_003239.4	GCGTGAGTGGCTGTTGAGA	AGGATTAGATGAGGGTTGTGGTG	Yes
TP53INP1	NM_033285.3	GCCCAAGTAGTCCCAGAGTG	AGTTGTATGAGCAGCAAGAGC	Yes
TYK2	NM_003331.4	AGCTTGTACTGCTACGATCCG	GACTTCTCGCCTTGGTCCTC	Yes
VIM	NM_003380.3	CAGATGCGTGAAATGGAAGA	TGGAAGAGGCAGAGAAATCC	Yes
ZMAT3	NM_022470.3	TATCGAAGGGAGGGGAGCAA	TTAAAGGAGCCCATCTGCGG	Yes
